# Potential Prion Involvement in Long COVID-19 Neuropathology, Including Behavior

**DOI:** 10.1007/s10571-023-01342-8

**Published:** 2023-03-28

**Authors:** George B. Stefano, Pascal Büttiker, Simon Weissenberger, Martin Anders, Jiri Raboch, Radek Ptacek, Richard M. Kream

**Affiliations:** 1 First Faculty of Medicine, Department of Psychiatry of the First Faculty of Medicine and General Teaching Hospital, Charles University in Prague, 120 00 Prague 2, Ke Karlovu 11, Prague, Czech Republic; 2 Department of Psychology, University of New York in Prague, 120 00 Prague 2, Londýnská 41, Prague, Czech Republic

**Keywords:** SARS-CoV-2, COVID-19, Long COVID, Prion, Prion disorders, Mitochondria, Confusion, Depression

## Abstract

Prion’ is a term used to describe a protein infectious particle responsible for several neurodegenerative diseases in mammals, e.g., Creutzfeldt-Jakob disease. The novelty is that it is protein based infectious agent not involving a nucleic acid genome as found in viruses and bacteria. Prion disorders exhibit, in part, incubation periods, neuronal loss, and induce abnormal folding of specific normal cellular proteins due to enhancing reactive oxygen species associated with mitochondria energy metabolism. These agents may also induce memory, personality and movement abnormalities as well as depression, confusion and disorientation. Interestingly, some of these behavioral changes also occur in COVID-19 and mechanistically include mitochondrial damage caused by SARS-CoV-2 and subsequenct production of reactive oxygen species. Taken together, we surmise, in part, long COVID may involve the induction of spontaneous prion emergence, especially in individuals susceptible to its origin may thus explain some of its manesfestions post-acute viral infection.

## Introduction

Prion diseases are a group of incurable neurodegenerative disorders that result from the accumulation of abnormally folded prion proteins in the brain. Although they contain no DNA or RNA, these abnormally folded proteins propagate as infectious agents and serve as templates for the conversion of protease-sensitive protein (PrP^C^) into protease-resistant, conformationally matched pathological isoforms (PrP^Sc^). Prion diseases (also known as transmissible spongiform encephalopathies, or TSEs) are uniformly fatal and have been diagnosed in humans and numerous animal species (Tian and Dong 2013). Creutzfeldt-Jakob disease (CJD), Gerstmann-Straussler-Scheinker disease, kuru, and fatal familial insomnia are among the disorders that have been identified as prion diseases (De Armond and Bouzamondo 2002; McKintosh et al. 2003).

## Discussion

### Role of Mitochondria in the Pathogenesis of Prion Diseases

By current estimates, ~ 1–2 million people worldwide are affected by prion diseases (Zambrano et al. 2022). These infectious and propagating prion proteins cluster in brain cells where they induce cell death and tissue degeneration. Mechanistically, prions induce pathologic changes in cellular metabolism and energy production via their capacity to damage mitochondria and impair mitochondrial function (Zambrano et al. 2022). Given their crucial role in maintaining the cellular energy supply, mitochondrial damage and subsequent dysfunction may be a critical first step in the pathogenesis of prion diseases (Zambrano et al. 2022). Importantly, mitochondrial proteins (e.g., mitofilin, heat shock protein and apoptosis-inducing factor) are coupled to prion-induced cell death (Moore et al. 2014). Furthermore, hamster with prion disease exhibit statistically significant decrease in mitochondrial respiration along with increased oxidative stress (Faris et al. 2017; Choi et al. 1998). Zambrano and colleagues (Zambrano et al. 2022) have hypothesized that interventions designed to preserve mitochondrial function might help cells to resist the rapid spread of these agents and the damage elicited by these misfolded prion proteins, and may even promote their clearance. Following this line of thought, we speculate that dysfunctional mitochondria may be more susceptible to infection and more effective at generating and propagating misfolded prion proteins, thereby contributing to the pathogenesis of prion disease.

Mitochondrial targeting and their increased sensitivity to reactive oxygen and reactive nitrogen species (ROS and RNS, respectively) underscore the role of these organelles in the pathogenesis of prion disorders. Overproduction and accumulation of both ROS and RNS, combined with an inadequate response from antioxidant enzyme systems, destroys cellular lipids, proteins, DNA, and RNA (Islam 2017; Benz et al. 2002), including those associated with mitochondria. The contributions of oxidative stress have been linked to the etiologies of numerous neurodegenerative diseases (NDDs), including Alzheimer’s disease, amyotrophic lateral sclerosis, Friedreich's ataxia, Huntington's disease, multiple sclerosis, and Parkinson's disease (Islam 2017; de la Torre and Stefano 2000). In addition, it can be surmised, ongoing oxidative stress may exacerbate protein misfolding and lead to other NDDs (Islam 2017; de la Torre and Stefano 2000).

Specifically, aberrant mitochondrial quality control (i.e., dysfunctional mitophagy) has been implicated as contributing to the pathogenesis of numerous human diseases, including cancer, cardiac dysfunction, and neurological disorders, notably prion disease (Kim et al. 2022). For example, Kim and colleagues (Kim et al. 2022). used scrapie-infected experimental models to explore the role of mitochondrial quality control in disease pathogenesis. Among their findings, they reported that scrapie infection led to the induction of mitochondrial reactive oxygen species (mtROS) and the loss of mitochondrial membrane potential (ΔΨm). These initial responses led to enhanced phosphorylation of dynamin-related protein 1 (Drp1) at Ser616 and followed by its translocation into the mitochondria followed by excessive mitophagy. Infection-associated aberrant mitochondrial fission and mitophagy also led to increases in apoptotic signaling, i.e., caspase 3 activation and poly (ADP-ribose) polymerase cleavage. These results suggest that scrapie infection led to impairments in mitochondrial quality control processes followed by neuronal cell death. Collectively, these mechanisms may play important roles in the neuropathogenesis of prion diseases.

### Prion Diseases and SARS-CoV-2

As per our current understanding, the misfolding of the cellular prion protein (PrP^C^) into its pathologic isoform (PrP^Sc^) is pathognomonic of primary prion disease (Hara et al. 2021). Interestingly, Hara and colleagues (Hara et al. 2021) performed a series of experiments that revealed that infection with a neurotropic strain of influenza A virus (IAV/WSN) resulted in the misfolding of PrP^C^ into PrP^Sc^ and the generation of infectious prions in mouse neuroblastoma cells. These results suggest that infection with an unrelated virus can induce misfolding of PrP^C^ into PrP^Sc^ and the formation of infectious prions. Recently, Young and colleagues (Young et al. 2020) described a man whose first manifestations of Creutzfeldt-Jakob disease (CJD) occurred in tandem with the symptomatic onset of Coronavirus disease-2019 (COVID-19). Drawing from recent findings focused on the pathogenesis of prion disease together with our current understanding of the immune responses to SARS-CoV-2, Young and colleagues (Young et al. 2020) hypothesized that the cascade of systemic inflammatory mediators synthesized and released in response to infection with SARS-CoV-2 serve to accelerate the development of pre-existing prion disease.

Recently, our group and others speculated on the nature of potential novel molecular neuropathological mechanisms associated with COVID-19, involving mitochondrial bioenergetics (Singh et al. 2020; Wu et al. 2020; Wang et al. 2020) and targeting of mitochondrial-mediated signaling pathways in response to the inflammatory sequelae of SARS-CoV-2 infection (Stefano and Kream 2022b; Stefano et al. 2022). It is of interest to note that DNA transfer from mitochondria to the eukaryotic cell genome represents an old evolutionary phenomenon, preceding human speciation (Wei et al. 2022). However, recent research by Wei and colleagues demonstrates, there is an ongoing transfer of mitochondrial DNA into the nuclear containing genome (nuclear-mitochondrial segments (NUMTs)). Furthermore, methylation processes inhibited the expression of this genetic material, however, some segments, a minority, are expressed. We speculate this common and old phenomenon maybe involved in the viral strategy of targeting mitochondria, leading to eukaryotic cell genome alteration and access whereby aberrant proteins emerge. Here, this phenomenon may become more evident behaviorally in neurons coupled to cognition since they are susceptible to a diminished energy supply.

Mitochondria are critical sources of ATP and are thus of fundamental importance in eukaryotic cells, notably those contributing to neural, cardiac, and immune system function. ATP is also required by the systems responsible for the clearance of pathological deposits, including amyloid-beta plaques in the brain that are characteristic of Alzheimer’s disease (Zattoni et al. 2022; Colini Baldeschi et al. 2022). Thus, the long-term neurological sequelae of SARS-CoV-2 infection might involve direct viral infection of mitochondria. Alternatively, virus infection may have an indirect impact on this organelle via a mechanism that results in long-term impairment and an inability to carry out its biological activities. The results of a recent computational modeling study revealed localized enrichment of genomic and subgenomic SARS-CoV-2 sequences, notably 5′ and 3′untranslated RNA sequences, within a host cell mitochondrial matrix as well as in nucleolar structures. The possibility that SARS-CoV-2 genetic material might reside in host mitochondria and potentially integrate into the host mitochondrial genome suggests that this virus may have direct access to the metabolic center of the cell and subvert the host metabolic system to conditions that are favorable for virus growth and replication (Stefano et al. 2021; Stefano and Kream 2022a; Singh et al. 2020). A mechanism involving viral control of mitochondrial metabolism might also account for the long-term neurological dysfunction that frequently results from SARS-CoV-2 infection. Infection of microglia may lead to impaired metabolic fitness and thus reductions in autophagy and metabolic support of basic functions, such as, clearance of pathologic plaques and deposits. Over the long term, virus-associated microglial dysfunction might lead to neurocognitive decline, which is among the emerging concepts in the pathophysiology of Alzheimer’s disease (Ulland et al. 2017; Stefano et al. 2020). It is important to recognize that viral hijacking of cellular metabolic function is not unique to SARS-CoV-2 or even coronaviruses. This mechanism has been proposed to explain the sequelae of other virus infections, including Ebola, Zika, and influenza A (Dutta et al. 2020). We hypothesize that mitochondrial dysfunction may also contribute to the pathogenesis of prion diseases.

In a recently published detailed analysis of the post-acute phase of COVID-19, Xu and colleagues (Xu et al. 2022) documented that individuals who had recovered from this disease were at an increased risk of numerous neurologic sequelae, including ischemic and hemorrhagic stroke, cognition and memory disorders, peripheral nervous system disorders, episodic disorders (e.g., migraine and seizures), extrapyramidal and movement disorders, mental health disorders, musculoskeletal disorders, sensory disorders, Guillain–Barré syndrome, and encephalitis/ encephalopathy, including those who did not require hospitalization for acute illness (Xu et al. 2022). Taken together, these findings provide evidence of an increased risk of long-term neurologic disorders in association with COVID-19.

In a recent report the estimate of human eukaryotic and bacterial cell levels was determined to be the same (approximately 10^13^), which occurs at the same concentration noted for viruses in equaling the total bacterial concentration (Liang and Bushman 2021; Shkoporov and Hill 2019). Considering the prokaryotic origin of mitochondria and that eukaryotic cells have the potential to harbor thousands of these basically distinct organelles one can surmise that the more “advanced cell” is highly dependent on entities that evolved much earlier in evolution. Thus, the simultaneous and interactive nature of evolution involving these entities emerges as more complex and diverse components of a eukaryotic cell’s life. Therefore, factors which modify and/or inhibit normal bacterial and viral “host” communication also would modify the eukaryotic cellular processes, contributing to an organism’s dysfunction. This also would explain the negative impact of nonindigenous microbes. Furthermore, since viruses, bacteria and eukaryotic cells, in part, communicate and direct the synthesis of proteins to carry out their reproductive associated strategies for existence, these proteins by chance could direct their own synthesis, that is bypass the need for nucleic acid direction, e.g., prions. We speculate prion represents a misstep in evolution given the potential of proteins to change shape and their spontaneous appearance under cell stress and stability for inter-organismic transfer and thus, emergence as pathological entities.

## Conclusion

**Fig. 1:**
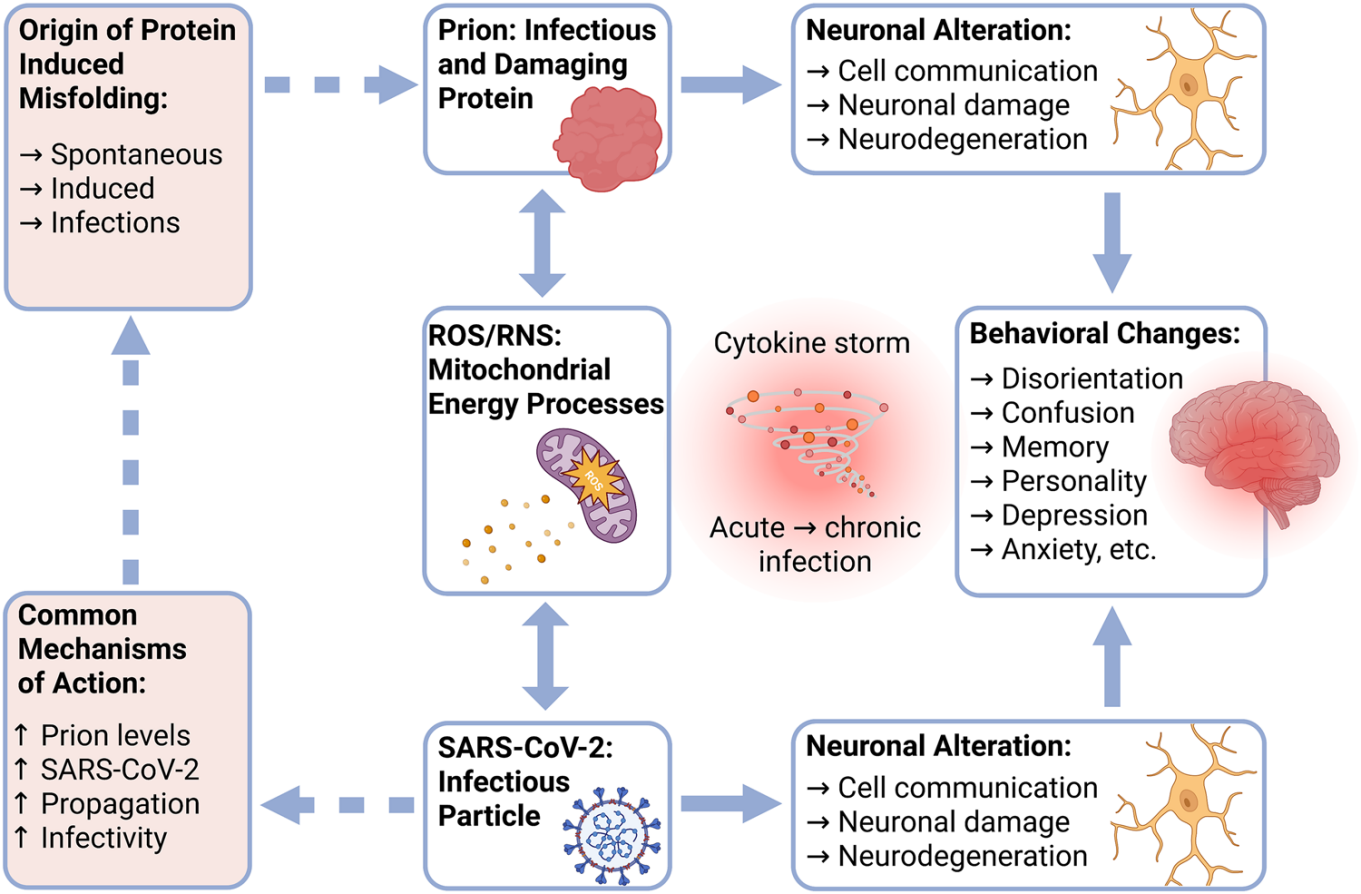
*Hypothetical mechanistic illustration of prion and SARS-CoV-2 mitochondrial targeting*. Prions are infectious proteinaceous particles that have been functionally associated with the progression of major human neurodegenerative disorders such as Creutzfeldt-Jakob disease. Empirically elucidated modes of actions of infectious prions include multiple inhibitory patterns adversely affecting normative neural communication as well as initiation of apoptotic or necrotic neural damage. Furthermore, a likely mechanism of prion-related neural dysfunction may also involve aberrant restitution of native conformation of abnormally folded cellular proteins. These pathophysiological effects may be potentially due to loss of normative proteasome functioning due to enhanced reactive oxygen species associated with compromised mitochondria energy metabolism. Infectious prion diseases may also induce memory, personality and movement abnormalities as well as depression, confusion and disorientation. Interestingly, some of these same behavioral sequelae have also been observed to occur subsequent to COVID-19 and may also share pathogenic mitochondrial damage caused by SARS-CoV-2 infection with subsequent production of ROS or RNS. Taken together, we speculate that pathophysiological effects of long COVID may involve the induction of spontaneous production of infectious prion species. Interestingly, mitochondria may represent the central focus of both induced disorders

Various viruses and virus infections can induce mitochondrial dysfunction. Thus, we hypothesize that the dysfunction of this bacterial-derived organelle is a critical component of many of these disorders. We proposed that this mechanism is directly related to the bacterial origin of this organelle at a time in evolutionary history that featured high rates of interaction between viruses and bacteria, with each serving as the primary target for one another. Current work suggests that there is a link between the pathophysiologic sequelae of SARS-CoV-2 infection and the pathogenesis of prion diseases. Specifically, SARS-CoV-2 contributes to the long-term pathological outcome of prion disease, i.e., neurodegeneration. Here, we propose a mechanism in which SARS-CoV-2 targets mitochondria and promotes their dysfunction. Likewise, SARS-CoV-2-mediated overproduction of ROS can lead to the misfolding of prion proteins that can then propagate in this environment, thereby accelerating the development of long-term pathology (Zhou et al. 2022; Yardeni et al. 2019) (Fig. 1). We note that certain tissues may be more susceptible to this mechanism, which may mask the true origin of the disease. In this regard, we hypothesize that illnesses that have been attributed to the syndrome known as long-term COVID-19 may actually originate, in part, from spontaneous prion production. We note that while fully developed prion disorders are universally fatal, misfolded proteins that accumulate in response to SARS-CoV-2 infection can most likely be cleared after a brief delay. By contrast, in the absence of another immunological challenge, the clearance mechanisms are severely overwhelmed in the setting of full-blown prion disease. Put simply, virus infection alters the process via which prions reproduce. Although SARS-CoV-2 has not yet been widely localized in the CNS, its’ damage has been associated with the infection (Douaud et al. 2022; Stein et al. 2022). This phenomenon warrants additional attention given the clinical similarities exhibited by long-term COVID-19 and prion diseases.

## Data Availability

Enquiries about data availability should be directed to the authors.
